# The association of spirometric small airways obstruction with respiratory symptoms, cardiometabolic diseases, and quality of life: results from the Burden of Obstructive Lung Disease (BOLD) study

**DOI:** 10.1186/s12931-023-02450-1

**Published:** 2023-05-23

**Authors:** Ben Knox-Brown, Jaymini Patel, James Potts, Rana Ahmed, Althea Aquart-Stewart, Cristina Barbara, A. Sonia Buist, Hamid Hacene Cherkaski, Meriam Denguezli, Mohammed Elbiaze, Gregory E. Erhabor, Frits M. E. Franssen, Mohammed Al Ghobain, Thorarinn Gislason, Christer Janson, Ali Kocabaş, David Mannino, Guy Marks, Kevin Mortimer, Asaad Ahmed Nafees, Daniel Obaseki, Stefanni Nonna M. Paraguas, Li Cher Loh, Abdul Rashid, Sundeep Salvi, Terence Seemungal, Michael Studnicka, Wan C. Tan, Emiel F. M. Wouters, Hazim Abozid, Alexander Mueller, Peter Burney, Andre F. S. Amaral

**Affiliations:** 1 National Heart and Lung Institute, Imperial College London, 1B Manresa Road, London, SW3 6LR UK; 2 The Epidemiological Laboratory (Epilab), Khartoum, Sudan; 3 Dept. of Medicine, UWI, Mona, Jamaica; 4 Faculdade de Medicina, Instituto de Saúde Ambiental, Universidade de Lisboa, Lisbon, Portugal; 5 Serviço de Pneumologia, Centro Hospitalar Universitário Lisboa Norte, Lisbon, Portugal; 6 Oregon Health & Science University, Portland, OR USA; 7 Dept of Pneumology, Faculty of Medicine Annaba, University Badji Mokhtar of Annaba, Annaba, Algeria; 8 Faculté de Médecine Dentaire de Monastir, Université de Monastir, Avenue Avicenne, Monastir, Tunisia; 9 Department of Respiratory Medicine, Faculty of Medicine, Mohammed Ben Abdellah University, University Hospital, Fes, Morocco; 10 Obafemi Awolowo University, Ile-Ife, Osun Nigeria; 11 Department of Respiratory Medicine, Maastricht University Medical Centre, Maastricht, The Netherlands; 12 Department of Research and Education, CIRO, Horn, the Netherlands; 13 King Abdullah International Medical Research Centre, King Saud Bin Abdulaziz University for Health Sciences, King Abdulaziz Medical City, Ministry of National Guard-Health Affairs, Riyadh, Saudi Arabia; 14 Department of Sleep, Landspitali University Hospital, Reykjavik, Iceland; 15 Faculty of Medicine, University of Iceland, Reykjavik, Iceland; 16 Department of Medical Sciences: Respiratory, Allergy and Sleep Research, Uppsala University, Uppsala, Sweden; 17 Department of Chest Diseases, Cukurova University School of Medicine, Adana, Turkey; 18 University of Kentucky, Lexington, KY USA; 19 COPD Foundation, Miami, FL USA; 20 Woolcock Institute of Medical Research, Sydney, NSW Australia; 21 University of Sydney, Sydney, NSW Australia; 22 University of New South Wales, Sydney, NSW Australia; 23 University of Cambridge, Cambridge, UK; 24 Liverpool University Hospitals NHS Foundation Trust, Liverpool, UK; 25 Department of Community Health Sciences, Aga Khan University, Karachi, Pakistan; 26 Philippine College of Chest Physicians, Quezon City, Philippines; 27 Philippine Heart Centre, Quezon City, Philippines; 28 RCSI & UCD Malaysia Campus, Penang, Malaysia; 29 Pulmocare Research and Education (PURE) Foundation, Pune, India; 30 Symbiosis International (Deemed University), Pune, India; 31 Faculty of Medical Sciences, University of the West Indies, St Augustine, Trinidad and Tobago; 32 University Clinic for Pneumology, Paracelsus Medical University Salzburg, Salzburg, Austria; 33 Centre for Heart Lung Innovation, University of British Columbia, Vancouver, BC Canada; 34 Ludwig Boltzmann Institute for Lung Health, Vienna, Austria

**Keywords:** Spirometry, Small airways obstruction, Symptoms, Cardiovascular disease, Quality of life

## Abstract

**Background:**

Spirometric small airways obstruction (SAO) is common in the general population. Whether spirometric SAO is associated with respiratory symptoms, cardiometabolic diseases, and quality of life (QoL) is unknown.

**Methods:**

Using data from the Burden of Obstructive Lung Disease study (N = 21,594), we defined spirometric SAO as the mean forced expiratory flow rate between 25 and 75% of the FVC (FEF_25-75_) less than the lower limit of normal (LLN) or the forced expiratory volume in 3 s to FVC ratio (FEV_3_/FVC) less than the LLN. We analysed data on respiratory symptoms, cardiometabolic diseases, and QoL collected using standardised questionnaires. We assessed the associations with spirometric SAO using multivariable regression models, and pooled site estimates using random effects meta-analysis. We conducted identical analyses for isolated spirometric SAO (i.e. with FEV_1_/FVC ≥ LLN).

**Results:**

Almost a fifth of the participants had spirometric SAO (19% for FEF_25-75_; 17% for FEV_3_/FVC). Using FEF_25-75,_ spirometric SAO was associated with dyspnoea (OR = 2.16, 95% CI 1.77–2.70), chronic cough (OR = 2.56, 95% CI 2.08–3.15), chronic phlegm (OR = 2.29, 95% CI 1.77–4.05), wheeze (OR = 2.87, 95% CI 2.50–3.40) and cardiovascular disease (OR = 1.30, 95% CI 1.11–1.52), but not hypertension or diabetes. Spirometric SAO was associated with worse physical and mental QoL. These associations were similar for FEV_3_/FVC. Isolated spirometric SAO (10% for FEF_25-75_; 6% for FEV_3_/FVC), was also associated with respiratory symptoms and cardiovascular disease.

**Conclusion:**

Spirometric SAO is associated with respiratory symptoms, cardiovascular disease, and QoL. Consideration should be given to the measurement of FEF_25-75_ and FEV_3_/FVC, in addition to traditional spirometry parameters.

**Supplementary Information:**

The online version contains supplementary material available at 10.1186/s12931-023-02450-1.

## Introduction

Spirometric small airways obstruction (SAO) is characterised by an airflow limitation through the mid to late portion of a maximal forced expiratory manoeuvre. It is most commonly defined by an abnormality in either the mean forced expiratory flow rate between 25 and 75% of the forced vital capacity (FEF_25-75_) or the forced expiratory volume in 3 s to forced vital capacity ratio (FEV_3_/FVC) [1]. Despite uncertainty as to its sensitivity and specificity [2], spirometric SAO is often used as a proxy for small airways disease, suggesting the presence of airflow limitation through airways of less than 2 mm diameter [3]. The small airways are integral in the pathophysiology of obstructive lung diseases such as asthma and chronic obstructive pulmonary disease (COPD), where inflammation, mucus hypersecretion, and airway remodelling are associated with increased respiratory symptoms, cardiometabolic complications, and reduced quality of life (QoL) [4–6]. Whether these associations are also seen with spirometric SAO in the general population, particularly in the absence of established lung disease is unknown.

Few studies have investigated spirometric SAO in general populations. Prevalence estimates range from 7.5% to 45.9%, influenced by the choice of spirometry parameter and world region [1]. Risk factors include active and passive smoking, low body mass index (BMI), increasing age, low education level, occupational exposure to dust, previous TB, and family history of COPD [7, 8]. There is now an increasing interest in understanding isolated spirometric SAO, which is characterised by the presence of spirometric SAO in the absence of established airflow limitation (i.e. with FEV_1_/FVC ≥ LLN). The reason for this is that some studies have reported an association between isolated spirometric SAO and early lung injury, including gas trapping and reduced diffusing capacity on lung function testing [9], as well as functional small airways disease and emphysema on quantitative chest CT [10–12]. There are also data suggesting that those with isolated spirometric SAO may be at increased risk of developing COPD [13].

As COPD has been associated with respiratory symptoms, cardiometabolic diseases and reduced QoL, it is reasonable to hypothesise that spirometric SAO may hold similar associations. However, current evidence is only available in ever smokers and not representative of the general population [11, 13]. With this in mind, we investigated these associations using data from the multinational Burden of Obstructive Lung Disease (BOLD) study and compared the findings for two different spirometry parameters.

## Methods

### Study design and participants

The design and rationale for the BOLD study have been previously published [14]. Non-institutionalised adults ≥ 40 years of age were recruited from 41 sites, across 34 countries, where population size was larger than 150,000. Standardised questionnaires were used to collect information on respiratory symptoms, health status, and exposure to potential risk factors. Questionnaires were translated into the local language and administered by trained fieldworkers. Measurements of height and weight were taken. Lung function was assessed before and 15 min after inhalation of 200mcg salbutamol, using the ndd EasyOne Spirometer (ndd Medizintechnik AG, Zurich, Switzerland). Spirometry parameters including the forced expiratory volume in 1 s (FEV_1_), FVC, FEV_3_, and FEF_25-75_ were measured. Spirograms were assigned quality scores based on the American Thoracic Society (ATS) acceptability and reproducibility criteria [15]. Quality was checked centrally, and only tests with back-extrapolated volume < 150 mL, peak expiratory flow time < 120 ms, lasting ≥ 6 s or with end-of-time volume < 40 mL, no artefact affecting the FEV_1_ or FVC, and with the two best blows within 200 mL of each other were used. A total of 28,604 participants had acceptable spirometry and completed the core questionnaire. Of these, 4573 were excluded as they did not have a measurement for both FEV_3_/FVC and FEF_25-75_. A further 2437 were excluded for not having complete information on respiratory symptoms, cardiometabolic diseases and QoL, leaving 21,594 participants for inclusion in the present study. Ethical approval was obtained by each site from the local ethics committee, all sites adhered to local ethics guidelines, and followed good clinical practice. Informed consent was obtained from all participants.

### Spirometric small airways obstruction

Due to lack of consensus in the literature [1], we defined spirometric SAO for two different spirometry parameters: (1) pre-bronchodilator FEF_25-75_ less than the lower limit of normal (LLN); and (2) pre-bronchodilator FEV_3_/FVC less than the LLN. We also defined airflow obstruction as pre-bronchodilator FEV_1_/FVC < LLN, and spirometric restriction as FVC < LLN. Additionally, we defined “isolated spirometric SAO” as FEF_25-75_ or FEV_3_/FVC less than the LLN with pre-bronchodilator FEV_1_/FVC equal or greater than the LLN. We used reference equations for European Americans in the third US National Health and Nutrition Examination Survey (NHANES) to calculate the LLN for all parameters [16, 17].

### Respiratory symptoms, cardiometabolic diseases, and QoL

Dyspnoea was assessed using the mMRC dyspnoea scale, where participants rated their breathlessness according to 5 grades: Grade 0—dyspnoea only with strenuous exercise; Grade 1—dyspnoea when hurrying on the level or up a slight hill; Grade 2—dyspnoea when walking at own pace on the level; Grade 3—dyspnoea when walking 100 yards or for a few minutes; Grade 4—too short of breath to leave the house or short of breath when dressing or undressing. We generated a binary variable where a grade of 0–1 indicates no/minimal breathlessness, and a grade ≥ 2 indicates significant breathlessness. Presence of chronic cough, chronic phlegm, and wheeze was determined by positive responses to the following questions: (1) *“do you cough on most days for as much as 3 months each year?”*; (2) “*do you bring up phlegm on most days for as much 3 months each year?”;* and (3)* “have you had wheezing or whistling in the chest at any time in the last 12 months?”.*

Information on self-reported, physician-diagnosed cardiometabolic diseases was obtained from the core study questionnaire. For the present analysis, we considered three outcomes: (1) cardiovascular disease (CVD) as the history of either heart disease or stroke; (2) history of hypertension; and (3) history of diabetes.

QoL was assessed using the 12-item short form health survey (SF-12). Separate scores for physical and mental health were generated and used in the analyses. Scores ranged from 0 to 100, with a score of 100 indicating the best QoL [18].

### Statistical analysis

To assess the association of respiratory symptoms with spirometric SAO, we used multivariable logistic regression analysis, adjusting for potential confounders [19]: sex, education level (none, primary or middle school, secondary school, and technical/vocational college or university), BMI (underweight < 18.5 kg m^−2^, normal 18.5–24.9 kg m^−2^, overweight 25–30 kg m^−2^ and obese > 30 kg m^−2^), age (40–49, 50–59, 60–69, ≥ 70 years), smoking status (never, former, current), smoking pack-years (1–5, 6–15, 16–25 or > 25), passive smoking, occupational exposure to dust ≥ 10 years, use of solid fuels for cooking/heating for > 6 months in a lifetime, history of tuberculosis, spirometric restriction and family history of COPD. For dyspnoea only, we added history of CVD into the model.

To assess the association of cardiometabolic diseases with spirometric SAO, we used multivariable logistic regression, adjusting for known cardiovascular risk factors [20–22]: sex, education level, BMI, age, smoking status, smoking pack-years and spirometric restriction.

To assess the association of QoL with spirometric SAO, we performed linear regression analysis using continuous proxies for physical and mental health scores. We adjusted for the same potential confounders as in the models for respiratory symptoms with the addition of CVD, hypertension, and diabetes.

We first assessed these associations within each site and then pooled their estimates using random effects meta-analyses [23]. We then repeated these analyses stratifying by sex. We performed sensitivity analyses repeating the analyses: (1) only among never smokers; (2) after excluding participants with both spirometric SAO and FEV_1_/FVC < LLN (isolated spirometric SAO); and (3) for symptoms and cardiometabolic diseases, using FEF_25-75_ and FEV_3_/FVC as continuous variables. For the association with cardiometabolic diseases, we also repeated the analyses among only those with a normal FVC. Heterogeneity was summarised using the I^2^ statistic. All analyses were performed using Stata 17 (Stata Corp., College Station, TX, USA) and corrected for sampling weights.

### Role of the funding source

The funders of the study did not contribute to the study design, data collection, data analysis or writing of the manuscript.

## Results

The characteristics of study participants are displayed in Table 1. The mean age of the participants was 54 years, with 51% being female. On average, they were slightly overweight (BMI 26.4 kg/m^2^), and two thirds had never smoked. Overall, a fifth of participants had spirometric SAO. For FEF_25-75_, prevalence ranged from 5% in Tartu (Estonia) to 33% in Mysore (India). Using FEV_3_/FVC, prevalence of spirometric SAO ranged from 5% in Riyadh (Saudi Arabia) to 30% in Salzburg (Austria). Prevalence of isolated spirometric SAO was lower, ranging from 1% in Tartu (Estonia) to 26% in Mysore (India) for FEF_25-75_, and from 1% in Riyadh (Saudi Arabia) to 14% in Bergen (Norway) for FEV_3_/FVC (Table 2). Approximately, one in ten participants had airflow obstruction, with a third having spirometric restriction.

**Table 1:** Characteristics of participants from 41 sites of the BOLD I (Burden of Obstructive Lung Disease) study with good quality spirometry measuring spirometric SAO and information on comorbidity, symptoms and QoL

BOLD Centre	*n*	M *n* (%)	Age, yr (mean, SD)	BMI (mean, SD)	Never smoked *n* (%)	CVD *n* (%)	Hypertension *n* (%)	Diabetes *n* (%)	Dyspnoea *n* (%)	Chronic cough *n* (%)	Chronic phlegm *n* (%)	Wheeze *n* (%)	Physical SF-12 (mean, SD)	Mental SF-12 (mean, SD)
Albania (Tirana)	832	416 (50%)	53.9 (10.6)	27.6 (4.2)	532 (64%)	17 (2%)	166 (20%)	58 (7%)	75 (9%)	75 (9%)	17 (2%)	33 (4%)	52.0 (6.3)	51.3 (5.7)
Algeria (Annaba)	802	401 (50%)	52.2 (10.0)	28.3 (5.6)	489 (61%)	48 (6%)	176 (22%)	112 (14%)	104 (13%)	24 (3%)	16 (2%)	112 (14%)	50.1 (8.4)	49.3 (7.2)
Australia (Sydney)	387	186 (48%)	58.4 (12.1)	27.7 (4.9)	190 (49%)	46 (12%)	120 (31%)	31 (8%)	27 (7%)	23 (6%)	19 (5%)	89 (23%)	48.9 (7.6)	51.5 (9.4)
Austria (Salzburg)	887	497 (56%)	57.0 (11.3)	26.3 (3.9)	408 (46%)	115 (13%)	275 (31%)	53 (6%)	62 (7%)	44 (5%)	80 (9%)	124 (14%)	50.8 (7.1)	54.4 (8.7)
Benin (Sémé-Kpodji)	488	224 (46%)	51.1 (9.6)	26.1 (5.3)	478 (98%)	15 (3%)	122 (25%)	10 (2%)	5 (1%)	5 (1%)	15 (3%)	5 (1%)	–	–
Cameroon (Limbe)	249	147 (59%)	51.8 (9.4)	26.6 (5.4)	192 (77%)	0 (0%)	27 (11%)	7 (3%)	15 (6%)	5 (2%)	0 (0%)	12 (5%)	–	–
Canada (Vancouver)	594	226 (38%)	55.3 (11.5)	26.5 (5.2)	255 (43%)	77 (13%)	107 (18%)	42 (7%)	48 (8%)	71 (12%)	65 (11%)	149 (25%)	51.6 (9.1)	50.7 (9.6)
China (Guangzhou)	295	156 (53%)	54.4 (10.0)	23.5 (3.2)	150 (51%)	27 (9%)	50 (17%)	6 (2%)	12 (4%)	27 (9%)	24 (8%)	6 (2%)	42.1 (7.7)	40.1 (7.2)
England (London)	539	253 (47%)	58.1 (11.3)	27.0 (4.7)	183 (34%)	43 (8%)	178 (33%)	38 (7%)	75 (14%)	70 (13%)	70 (13%)	205 (38%)	50.0 (9.4)	50.0 (11.0)
Estonia (Tartu)	545	262 (48%)	59.7 (11.6)	28.3 (5.3)	294 (54%)	185 (34%)	202 (37%)	33 (6%)	71 (13%)	38 (7%)	44 (8%)	114 (21%)	47.1 (9.0)	52.3 (8.6)
Germany (Hannover)	414	224 (54%)	57.2 (10.7)	26.8 (4.3)	161 (39%)	58 (14%)	137 (33%)	21 (5%)	17 (4%)	37 (9%)	29 (7%)	58 (14%)	48.7 (8.2)	56.1 (7.5)
Iceland (Reykjavik)	552	298 (54%)	56.1 (11.3)	27.7 (16.6)	182 (33%)	88 (16%)	177 (32%)	22 (4%)	44 (8%)	61 (11%)	50 (95)	127 (23%)	50.6 (7.8)	53.6 (8.8)
India Mumbai	315	208 (66%)	51.0 (8.9)	23.9 (4.0)	280 (89%)	6 (2%)	28 (9%)	19 (6%)	25 (8%)	3 (1%)	3 (1%)	9 (3%)	53.1 (6.3)	58.5 (6.4)
India (Mysore)	531	228 (43%)	46.3 (6.8)	24.6 (3.6)	483 (91%)	0 (0%)	90 (17%)	85 (16%)	0 (0%)	5 (1%)	11 (2%)	0 (0%)	53.3 (4.1)	58.3 (7.2)
India (Pune)	732	439 (60%)	52.2 (9.7)	22.1 (3.9)	637 (87%)	15 (2%)	37 (5%)	15 (2%)	44 (6%)	15 (2%)	7 (1%)	29 (4%)	50.2 (6.4)	49.3 (7.3)
India (Kashmir)	677	366 (54%)	51.2 (10.3)	22.4 (3.6)	325 (48%)	7 (1%)	183 (27%)	20 (3%)	34 (5%)	41 (6%)	41 (6%)	20 (3%)	51.4 (6.6)	51.7 (6.3)
Jamaica	459	202 (44%)	55.1 (11.2)	27.5 (6.5)	280 (61%)	9 (2%)	129 (28%)	46 (10%)	60 (13%)	23 (5%)	18 (4%)	73 (16%)	–	–
Kyrgyzstan (Chui)	738	251 (34%)	52.5 (8.5)	28.0 (5.3)	450 (61%)	103 (14%)	177 (24%)	37 (5%)	118 (16%)	111 (15%)	74 (10%)	103 (14%)	–	–
Kyrgyzstan (Naryn)	755	294 (39%)	52.8 (9.6)	26.8 (4.8)	521 (69%)	83 (11%)	106 (14%)	8 (1%)	128 (17%)	68 (9%)	53 (7%)	98 (13%)	–	–
Malawi (Blantyre)	331	132 (40%)	51.8 (9.6)	24.7 (5.1)	275 (83%)	10 (3%)	53 (16%)	20 (6%)	7 (2%)	7 (2%)	0 (0%)	17 (5%)	53.7 (4.2)	53.5 (8.5)
Malawi (Chikwawa)	366	194 (53%)	53.1 (10.1)	21.7 (3.8)	260 (71%)	4 (1%)	7 (2%)	4 (1%)	4 (1%)	4 (1%)	0 (0%)	11 (3%)	52.1 (4.1)	55.9 (8.1)
Malaysia (Penang)	616	308 (50%)	54.4 (9.5)	26.1 (4.6)	462 (75%)	18 (3%)	154 (25%)	80 (13%)	62 (10%)	25 (4%)	25 (4%)	37 (6%)	–	–
Morocco (Fes)	429	240 (56%)	53.9 (9.9)	27.3 (4.9)	279 (65%)	13 (3%)	94 (22%)	43 (10%)	69 (16%)	26 (6%)	26 (6%)	39 (9%)	50.0 (9.1)	42.3 (8.4)
Netherlands (Maastricht)	538	274 (51%)	57.2 (10.5)	27.5 (4.5)	194 (36%)	91 (17%)	156 (29%)	38 (7%)	54 (10%)	27 (5%)	16 (3%)	102 (19%)	50.6 (8.6)	53.3 (9.5)
Nigeria (Ife)	793	317 (40%)	54.6 (11.6)	25.3 (5.3)	674 (85%)	0 (0%)	16 (2%)	8 (1%)	24 (3%)	0 (0%)	0 (0%)	16 (2%)	45.1 (8.5)	50.0 (9.9)
Norway (Bergen)	447	215 (48%)	58.3 (12.2)	26.3 (4.2)	148 (33%)	54 (12%)	112 (25%)	18 (4%)	22 (5%)	31 (7%)	40 (9%)	98 (22%)	50.3 (9.0)	54.4 (9.1)
Pakistan (Karachi)	311	162 (52%)	51.0 (9.0)	26.4 (5.9)	215 (69%)	9 (3%)	65 (21%)	31 (10%)	84 (27%)	28 (9%)	31 (10%)	28 (9%)	–	–
Philippines (Manila)	651	267 (41%)	52.1 (10.1)	24.9 (4.7)	286 (44%)	59 (9%)	150 (23%)	26 (4%)	150 (23%)	39 (6%)	78 (12%)	111 (17%)	46.8 (7.4)	53.6 (9.2)
Philippines (Nampicuan-Talugtug)	638	319 (50%)	54.0 (10.4)	21.5 (4.0)	287 (45%)	57 (9%)	134 (21%)	13 (2%)	172 (27%)	51 (8%)	70 (11%)	191 (30%)	46.4 (7.1)	50.5 (7.2)
Poland (Krakow)	336	171 (51%)	54.2 (10.6)	27.6 (4.8)	124 (37%)	101 (30%)	134 (40%)	37 (11%)	87 (26%)	27 (8%)	24 (7%)	94 (28%)	43.7 (11.0)	47.7 (10.1)
Portugal (Lisbon)	548	263 (48%)	62.2 (11.3)	28.0 (4.5)	307 (56%)	55 (10%)	159 (29%)	49 (9%)	71 (13%)	55 (10%)	55 (10%)	132 (24%)	48.9 (8.9)	50.0 (11.6)
Saudi Arabia (Riyadh)	576	323 (56%)	49.3 (7.3)	30.9 (5.8)	420 (73%)	29 (5%)	138 (24%)	156 (27%)	132 (23%)	63 (11%)	69 (12%)	225 (39%)	48.7 (8.5)	50.2 (8.8)
South Africa (Uitsig and Ravensmead)	505	187 (37%)	53.2 (9.7)	28.0 (7.8)	162 (32%)	51 (10%)	172 (34%)	56 (11%)	141 (28%)	51 (10%)	71 (14%)	136 (27%)	46.9 (9.1)	49.0 (10.2)
Sri Lanka (Colombo)	718	337 (47%)	53.1 (9.1)	24.4 (4.7)	539 (75%)	36 (5%)	144 (20%)	101 (14%)	194 (27%)	43 (6%)	57 (8%)	194 (27%)	–	–
Sudan (Gezeira)	277	172 (62%)	52.4 (9.3)	26.7 (17.3)	199 (72%)	0 (0%)	25 (9%)	17 (6%)	22 (8%)	6 (2%)	8 (3%)	36 (13%)	47.4 (7.2)	54.5 (9.0)
Sudan (Khartoum)	367	209 (57%)	53.5 (10.5)	26.6 (6.9)	294 (80%)	7 (2%)	77 (21%)	29 (8%)	26 (7%)	11 (3%)	11 (3%)	22 (6%)	48.9 (7.6)	48.2 (9.4)
Sweden (Uppsala)	393	204 (52%)	57.8 (10.9)	26.7 (4.2)	157 (40%)	43 (11%)	98 (25%)	12 (3%)	20 (5%)	28 (7%)	39 (10%)	90 (23%)	49.2 (6.3)	43.5 (6.8)
Trinidad & Tobago (Port of Spain)	815	334 (41%)	53.3 (10.2)	28.9 (10.5)	579 (71%)	41 (5%)	212 (26%)	114 (14%)	65 (8%)	57 (7%)	24 (3%)	82 (10%)	48.8 (6.2)	55.2 (8.6)
Tunisia (Sousse)	422	245 (58%)	51.6 (9.0)	28.1 (5.3)	198 (47%)	17 (4%)	59 (14%)	30 (7%)	63 (15%)	34 (8%)	51 (12%)	84 (20%)	47.6 (8.6)	50.3 (9.9)
Turkey (Adana)	414	215 (52%)	52.0 (9.4)	29.0 (5.1)	174 (42%)	41 (10%)	87 (21%)	29 (7%)	83 (20%)	29 (7%)	33 (8%)	137 (33%)	43.5 (8.2)	33.7 (7.7)
USA (Lexington)	312	122 (39%)	55.2 (9.7)	30.3 (6.7)	115 (37%)	72 (23%)	137 (44%)	41 (13%)	56 (18%)	53 (17%)	44 (14%)	134 (43%)	46.0 (11.0)	50.2 (11.0)

M: Male. BMI: body mass index. CVD: cardiovascular disease (stroke or heart disease). QoL: quality of life. n: subjects with acceptable pre-bronchodilator spirometry and complete information on characteristics, comorbidity, symptoms and QoL. Dyspnoea measured according to mMRC Dyspnoea scale: 0–1 = minimal/no breathlessness, ≥ 2 = significant breathlessness. Presence of chronic cough, chronic phlegm, and wheeze was determined by positive responses to the following questions: 1) “do you cough on most days for as much as 3 months each year?”; 2) “do you bring up phlegm on most days for as much 3 months each year?”; and 3) “have you had wheezing or whistling in the chest at any time in the last 12 months?”. Physical and mental QoL measured using the SF-12 questionnaire. Sites with missing data for SF-12 did not use this tool to assess QoL

**Table 2:** Summary of prevalence estimates for airflow obstruction, restriction, and spirometric SAO in the study population

BOLD Centre	n	Airflow obstruction *n* (%)	Restriction *n* (%)	Spirometric SAO (FEV_3_/FVC) *n* (%)	Isolated spirometric SAO (FEV_3_/FVC) *n* (%)	Spirometric SAO (FEF_25-75_) *n* (%)	Isolated spirometric SAO (FEF_25-75_) *n* (%)
Albania (Tirana)	832	82 (10%)	123 (15%)	85 (10%)	22 (3%)	69 (8%)	22 (3%)
Algeria (Annaba)	802	85 (11%)	219 (27%)	75 (9%)	21 (3%)	103 (13%)	54 (7%)
Australia (Sydney)	387	58 (15%)	47 (12%)	72 (19%)	25 (7%)	43 (11%)	11 (3%)
Austria (Salzburg)	887	222 (25%)	74 (8%)	271 (31%)	114 (13%)	138 (16%)	25 (3%)
Benin (Sémé-Kpodji)	488	55 (11%)	385 (79%)	68 (14%)	36 (7%)	147 (30%)	110 (23%)
Cameroon (Limbe)	249	14 (6%)	151 (61%)	16 (7%)	7 (3%)	33 (13%)	23 (9%)
Canada (Vancouver)	594	105 (18%)	43 (7%)	135 (23%)	59 (10%)	69 (12%)	17 (3%)
China (Guangzhou)	295	33 (11%)	84 (29%)	37 (13%)	17 (6%)	54 (18%)	31 (11%)
England (London)	539	119 (22%)	83 (15%)	163 (30%)	70 (13%)	99 (18%)	31 (6%)
Estonia (Tartu)	545	69 (13%)	45 (8%)	83 (15%)	39 (7%)	29 (5%)	8 (1%)
Germany (Hannover)	414	55 (13%)	22 (5%)	53 (13%)	19 (5%)	36 (9%)	10 (2%)
Iceland (Reykjavik)	552	116 (21%)	69 (13%)	158 (29%)	63 (12%)	94 (17%)	25 (5%)
India (Mumbai)	315	26 (8%)	205 (65%)	27 (9%)	9 (3%)	68 (22%)	48 (15%)
India (Mysore)	531	49 (9%)	412 (78%)	55 (10%)	24 (5%)	176 (33%)	140 (26%)
India (Pune)	732	65 (9%)	474 (65%)	81 (11%)	36 (5%)	173 (24%)	130 (18%)
India (Kashmir)	677	144 (21%)	190 (28%)	156 (23%)	45 (7%)	179 (26%)	73 (11%)
Jamaica	459	45 (10%)	253 (55%)	84 (18%)	48 (10%)	87 (19%)	53 (12%)
Kyrgyzstan (Chui)	738	141 (19%)	81 (11%)	157 (215)	35 (5%)	137 (19%)	25 (3%)
Kyrgyzstan (Naryn)	755	84 (11%)	77 (10%)	124 (16%)	58 (8%)	108 (14%)	51 (7%)
Malawi (Blantyre)	331	33 (10%)	146 (44%)	58 (18%)	31 (10%)	82 (25%)	57 (17%)
Malawi (Chikwawa)	366	66 (18%)	135 (37%)	79 (22%)	30 (8%)	108 (30%)	64 (18%)
Malaysia (Penang)	616	25 (4%)	357 (58%)	38 (6%)	21 (3%)	78 (13%)	59 (10%)
Morocco (Fes)	429	37 (95)	80 (19%)	48 (11%)	15 (3%)	58 (14%)	31 (7%)
Netherlands (Maastricht)	538	124 (23%)	51 (9%)	151 (28%)	55 (10%)	95 (18%)	18 (3%)
Nigeria (Ife)	793	98 (12%)	568 (72%)	152 (19%)	76 (10%)	216 (27%)	149 (19%)
Norway (Bergen)	447	82 (18%)	39 (9%)	115 (265)	60 (14%)	66 (15%)	21 (5%)
Pakistan (Karachi)	311	38 (12%)	238 (77%)	49 (16%)	23 (7%)	86 (28%)	57 (18%)
Philippines (Manila)	651	98 (15%)	404 (62%)	89 (14%)	27 (4%)	154 (24%)	90 (14%)
Philippines (Nampicuan-Talugtug)	638	126 (20%)	366 (57%)	127 (20%)	40 (6%)	191 (30%)	112 (18%)
Poland (Krakow)	336	64 (19%)	34 (10%)	81 (24%)	33 (10%)	62 (19%)	20 (6%)
Portugal (Lisbon)	548	57 (10%)	52 (10%)	90 (165)	41 (7%)	54 (10%)	16 (3%)
Saudi Arabia (Riyadh)	576	31 (5%)	293 (51%)	29 (5%)	7 (1%)	63 (11%)	34 (6%)
South Africa (Uitsig and Ravensmead)	505	116 (23%)	232 (46%)	132 (26%)	46 (9%)	159 (31%)	79 (16%)
Sri Lanka (Colombo)	718	77 (11%)	602 (84%)	65 (9%)	12 (25)	200 (28%)	146 (20%)
Sudan (Gezeira)	277	20 (7%)	153 (55%)	30 (11%)	13 (5%)	38 (14%)	21 (8%)
Sudan (Khartoum)	367	39 (11%)	255 (70%)	56 (15%)	25 (7%)	76 (21%)	50 (14%)
Sweden (Uppsala)	393	56 (14%)	30 (8%)	56 (14%)	19 (5%)	31 (8%)	11 (3%)
Trinidad & Tobago (Port of Spain)	815	59 (7%)	606 (74%)	112 (14%)	72 (9%)	167 (21%)	121 (15%)
Tunisia (Sousse)	422	29 (7%)	98 (23%)	34 (8%)	16 (4%)	44 (10%)	22 (5%)
Turkey (Adana)	414	82 (20%)	56 (14%)	114 (28%)	53 (13%)	89 (22%)	33 (8%)
USA (Lexington)	312	51 (17%)	72 (23%)	55 (18%)	20 (6%)	69 (22%)	32 (10%)

Spirometric SAO: small airways obstruction—Pre-bronchodilator FEF_25-75_ or FEV_3_/FVC less than the lower limit of normal (LLN). Isolated spirometric SAO: Pre-bronchodilator FEF_25-75_ or FEV_3_/FVC less than the lower limit of normal with FEV_1_/FVC ≥ LLN. AO: airflow obstruction—pre-bronchodilator FEV_1_/FVC < LLN. Restriction: Post-bronchodilator FVC < LLN. LLN calculated using spirometry reference equations taken from the NHANES III study population. FVC: Forced vital capacity. FEF_25-75_: Mean forced expiratory flow rate between 25 and 75% of the FVC. FEV_3_/FVC: Forced expiratory volume in 3 s as a ratio of the FVC. FEV_1_/FVC: Forced expiratory volume in 1 s as a ratio of the FVC

The prevalence of respiratory symptoms and cardiometabolic diseases also varied: dyspnoea from 0% in Mysore (India) to 28% in Uitsig and Ravensmead (South Africa); chronic cough from 0% in Ife (Nigeria) to 17% in Lexington (KY, USA); chronic phlegm from 0% in both sites in Malawi to 14% in Lexington (KY, USA); wheeze from 0% in Mysore (India) to 43% in Lexington (KY, USA); CVD from 0% in Gezeira (Sudan), Mysore (India), and Limbe (Cameroon) to 34% in Tartu (Estonia); hypertension from 2% in Ife (Nigeria) to 44% in Lexington (KY, USA); and diabetes from 1% in Ife (Nigeria) to 27% in Riyadh (Saudi Arabia) (Table 1).

Physical QoL scores were lowest (mean 42.1, SD 7.7) in Guangzhou (China) and highest (mean 53.7, SD 4.2) in Blantyre (Malawi). Mental QoL scores were lowest (mean 33.7, SD 7.7) in Adana (Turkey) and highest (mean 58.5, SD 6.4) in Mumbai (India) (Table 1).

### Respiratory symptoms and spirometric SAO

**Fig. 1:**
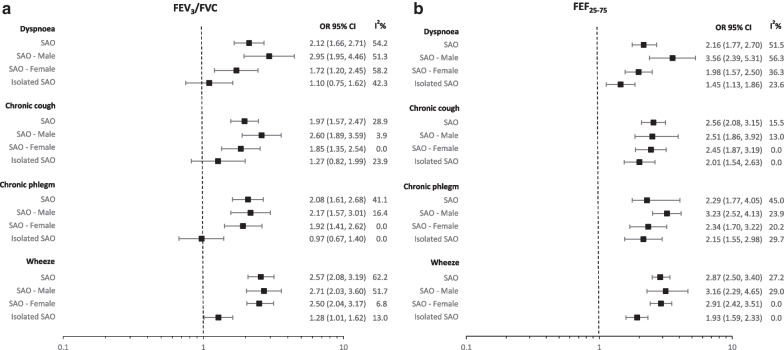
Pooled estimates for the effect of spirometric SAO measured using FEV_3_/FVC (**a**) and FEF_25-75_ (**b**) on respiratory symptoms in the BOLD study. Spirometric SAO: Small airways obstruction. Overall: spirometric SAO defined as FEV_3_/FVC or FEF_25-75_ less than the lower limit of normal (LLN). Male/Female: spirometric SAO as FEV_3_/FVC or FEF_25-75_ < LLN stratified by sex. Isolated spirometric SAO: FEV_3_/FVC or FEF_25-75_ < LLN with FEV_1_/FVC ≥ LLN. Dyspnoea measured according to mMRC Dyspnoea scale: 0–1 = minimal/no breathlessness, ≥ 2 = significant breathlessness. Chronic cough: cough on most days for 3 months each year. Chronic Phlegm: Phlegm on most days 3 months each year. Wheeze: Wheezing or whistling in the chest at any time in the last 12 months. OR 95% CI: odds ratio with 95% confidence intervals. I^2^ values of 0%, 25%, 50%, and 75% considered no, low, moderate, and high heterogeneity. Covariates in the adjusted model: sex, education level, body mass index, smoking status, accumulated cigarette pack-years, passive smoking, occupational exposure to dust, use of solid fuels for cooking/heating for > 6 months in a lifetime, reported doctor-diagnosed or history of tuberculosis, spirometric restriction, family history of COPD, and for Dyspnoea addition of CVD. The following sites could not be included in the analysis either due to a low number of participants reporting respiratory symptoms or singularity in the data: Benin (Sémé-Kpodji), Norway (Bergen), Malawi (Blantyre), China (Guangzhou), Germany (Hannover), Cameroon (Limbe), India (Mumbai) (Mysore), Austria (Salzburg), Tunisia (Sousse), Australia (Sydney), Albania (Tirana), Sweden (Uppsala)

Participants with spirometric SAO, based on FEF_25-75_, were more likely to report dyspnoea (OR = 2.16, 95% CI 1.77–2.70), chronic cough (OR = 2.56, 95% CI 2.08–3.15), chronic phlegm (OR = 2.29, 95% CI 1.77–4.05), and wheeze (OR = 2.87, 95% CI 2.50–3.40) than those without spirometric SAO (Fig. 1a and b; Additional file 1: Table S1). Associations were slightly stronger among males.

Among never smokers, spirometric SAO based on either FEF_25-75_ or FEV_3_/FVC was still associated with increased odds of all respiratory symptoms for both parameters (Additional file 1: Table S4). Results for spirometric SAO based on FEV_3_/FVC were not materially different from these, except when considering only isolated spirometric SAO, which was associated with all respiratory symptoms for FEF_25-75_ but only wheeze when using FEV_3_/FVC (Fig. 1a and b). Heterogeneity across sites for the association of spirometric SAO with respiratory symptoms was generally low-moderate. The association of post-bronchodilator spirometric SAO with respiratory symptoms was not materially different from those with pre-bronchodilator spirometric SAO. Overall, respiratory symptoms were associated with FEF_25-75_ and FEV_3_/FVC in a dose–response manner (Additional file 1: Tables S6 and S7).

### Cardiometabolic diseases and spirometric SAO

**Fig. 2:**
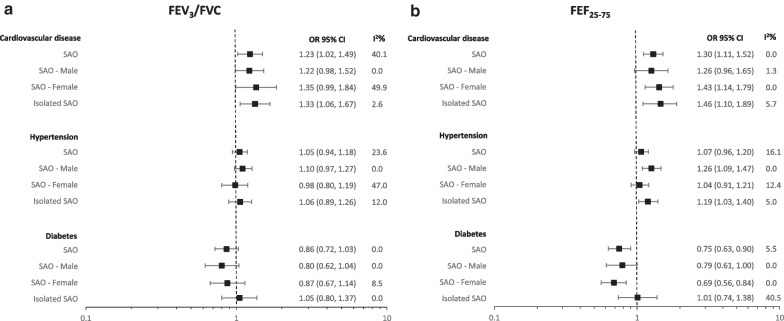
Pooled estimates for the effect of spirometric SAO measured using FEV_3_/FVC (**a**) and FEF_25-75_ (**b**) on cardiometabolic disease in the BOLD study. Spirometric SAO: Small airways obstruction. Overall: spirometric SAO defined as FEV_3_/FVC or FEF_25-75_ less than the lower limit of normal (LLN). Male/Female: spirometric SAO as FEV_3_/FVC or FEF_25-75_ < LLN stratified by sex. Isolated spirometric SAO: FEV_3_/FVC or FEF_25-75_ < LLN with FEV_1_/FVC ≥ LLN. Cardiovascular disease: self-reported history of heart disease or stroke. OR (95% CI): odds ratio with 95% confidence intervals. I^2^ values of 0%, 25%, 50%, and 75% considered no, low, moderate, and high heterogeneity. Covariates in the adjusted model: sex, education level, body mass index, smoking status, accumulated cigarette pack-years and spirometric restriction. The following sites could not be included in the analysis either due to a low number of participants reporting co-morbidity or singularity in the data: For CVD; Malawi (Blantyre), Malawi (Chikwawa), Nigeria (Ife), Cameroon (Limbe), India (Mysore), India (Kashmir), Malaysia (Penang), Sudan (Gezeira), Morocco (Fes), China (Guangzhou), Jamaica, Trinidad & Tobago (Port of Spain), Saudi Arabia (Riyadh), and Albania (Tirana). For Hypertension; Benin (Sémé-Kpodji), Malawi (Chikwawa), and Sudan (Gezeira). For diabetes; India (Pune), Malawi (Chikwawa), Morocco (Fes), China (Guangzhou), Nigeria (Ife), Kyrgyzstan (Naryn), Cameroon (Limbe), Philippines (Manilla), India (Mumbai), Malaysia (Penang)

Participants with spirometric SAO, based on FEF_25-75_, were more likely to have CVD (OR = 1.30, 95% CI 1.11–1.52) but less likely to have diabetes (OR = 0.75, 95% CI 0.63, 0.90), as compared to those without spirometric SAO. Overall, spirometric SAO was not associated with a diagnosis of hypertension (OR = 1.07, 95% CI 0.96, 1.20) (Fig. 2a and b; Additional file 1: Table S2). Associations did not differ much by sex. Results for spirometric SAO based on FEV_3_/FVC were not materially different from these.

Among never smokers only, spirometric SAO based on FEF_25-75_ was associated with CVD (OR = 1.45 95% CI 1.15–1.82) but not hypertension (OR = 1.10, 95% CI 0.97–1.25) or diabetes (OR = 0.84, 95% CI 0.69–1.02). Based on FEV_3_/FVC, spirometric SAO was not associated with any of the three cardiometabolic diseases (Additional file 1: Table S4).

In a sensitivity analysis in which those with low FVC were excluded, spirometric SAO based on FEF_25-75_ was associated with CVD (OR = 1.38, 95% CI 1.13–1.68) and hypertension (OR = 1.22, 95% CI 1.08–1.39) but not diabetes (OR = 0.86, 95% CI 0.67–1.11). Based on FEV_3_/FVC, spirometric SAO was not associated with any of the three cardiometabolic diseases (Additional file 1: Table S5).

Isolated spirometric SAO, based on either FEF_25-75_ or FEV_3_/FVC, was associated with CVD but not diabetes. The association with hypertension was not concordant between the two parameters used to define isolated spirometric SAO. Heterogeneity across sites was low for all estimates. The association of post-bronchodilator spirometric SAO with comorbidities was not materially different from those with pre-bronchodilator spirometric SAO. CVD was associated with FEF_25-75_ and FEV_3_/FVC in a dose–response manner (Additional file 1: Tables S6 and S7).

### Quality of life and spirometric SAO

**Fig. 3:**
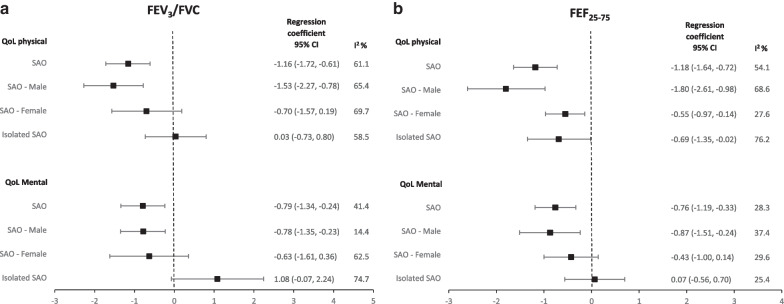
Pooled estimates for the effect of spirometric SAO measured using FEV_3_/FVC (**a**) and FEF_25-75_ (**b**) on physical and mental quality of life in the BOLD study. Spirometric SAO: Small airways obstruction. Overall: spirometric SAO defined as FEV_3_/FVC or FEF_25-75_ less than the lower limit of normal (LLN). Male/Female: spirometric SAO as FEV_3_/FVC or FEF_25-75_ < LLN stratified by sex. Isolated spirometric SAO: FEV_3_/FVC or FEF_25-75_ < LLN with FEV_1_/FVC ≥ LLN. Physical and mental QoL measured using the SF-12 questionnaire. Negative regression coefficient indicates that having SAO is associated with a reduction in SF-12 score in comparison to not having SAO. I^2^ values of 0%, 25%, 50%, and 75% considered no, low, moderate, and high heterogeneity. Covariates in the adjusted model: sex, education level, body mass index, smoking status, accumulated cigarette pack-years, passive smoking, occupational exposure to dust, use of solid fuels for cooking/heating for > 6 months in a lifetime, reported doctor-diagnosed or history of tuberculosis, spirometric restriction, family history of COPD, CVD, hypertension, and diabetes. Estimates based on the analysis of 31 sites, the following sites could not be included in the analysis either due to low response rate to the questionnaire; Turkey (Adana) and China (Guangzhou) or where QoL was measured using a different tool; Benin (Sémé-Kpodji), Cameroon (Limbe), Jamaica, Kyrgyzstan (Chui), Kyrgyzstan (Naryn), Malaysia (Penang), Pakistan (Karachi), Sri Lanka (Colombo)

Participants with spirometric SAO, based on FEF_25-75_, were more likely to show lower physical (β = − 1.18, 95% CI − 1.64 to − 0.72) and mental (β = − 0.76, 95% CI − 1.19 to − 0.33) scores of QoL (Fig. 3a and b; Additional file 1: Table S3).

The association of QoL, particularly of the physical component, with spirometric SAO was stronger among males than among females. Results for spirometric SAO based on FEV_3_/FVC were not materially different from these.

When we restricted our analyses to never smokers, lower physical and mental QoL was still associated with spirometric SAO based on either FEF_25-75_ or FEV_3_/FVC (Additional file 1: Table S4).

A lower physical score was weakly associated with isolated spirometric SAO based on FEF_25-75_ (β = − 0.69, 95% CI − 1.35 to − 0.02), but not with isolated spirometric SAO based on FEV_3_/FVC (β = 0.03, 95% CI − 0.73 to 0.80). There was no evidence of association of mental QoL score with isolated spirometric SAO. Heterogeneity across sites was low to moderate for all estimates. The association of post-bronchodilator spirometric SAO with QoL was not materially different from those with pre-bronchodilator spirometric SAO.

## Discussion

In this multinational population-based study of adults, aged 40 years and above, we show that people with spirometric SAO are more likely to report dyspnoea, chronic cough, chronic phlegm, and wheeze. Additionally, they are more likely to have had a diagnosis of cardiovascular disease, but not hypertension or diabetes. A worse quality of life is also associated with spirometric SAO. All these findings are true also for people with spirometric SAO without airflow obstruction, except in terms of quality of life.

### Respiratory symptoms

Both obstructive and restrictive lung patterns have been associated with respiratory symptoms [19, 24], therefore our finding of associations between spirometric SAO and increased dyspnoea, chronic cough, chronic phlegm, and wheeze is not surprising. That said, in the present study, we also found associations between isolated spirometric SAO and respiratory symptoms, especially when using FEF_25-75_. Only two previous studies have reported associations between isolated spirometric SAO and respiratory symptoms. Yee et al. [13] in the SPIROMICS and Dilektasli et al. [11] in the COPDGene cohorts showed associations between isolated spirometric SAO and increased respiratory exacerbations and dyspnoea. However, unlike the BOLD study where never smokers make up a considerable proportion of the study population, these studies only included current or former smokers. Therefore, our study presents the first population-based evidence on the association of respiratory symptoms with spirometric SAO. This supports the hypothesis that spirometric SAO is a precursor to future airflow obstruction [11, 13], presenting an alternative avenue of investigation for clinicians, if traditional measurement indices do not explain the presence of symptoms.

Using FEF_25-75_, we found that isolated spirometric SAO associates with all respiratory symptoms, while isolated spirometric SAO defined using the FEV_3_/FVC was only associated with wheeze. A potential explanation for this is that unlike the FEF_25-75_, the FEV_3_/FVC also includes the volume expired in the first 25% of expiration. This volume comes predominantly from emptying of the large conducting airways, which are less likely to be impacted in mild disease. Conversely, the FEF_25-75_ is specific to the average rate of flow through the middle 50% of expiration, and possibly more sensitive to early changes in the small airways.

There are several potential mechanisms by which isolated spirometric SAO may lead to respiratory symptoms. Chronic exposure to inhaled irritants such as cigarette smoke, damages the walls of the small airways, which has been shown to occur even before airflow obstruction becomes evident [11, 13]. Hospital-based studies have shown that individuals with isolated spirometric SAO according to FEV_3_/FVC have impaired diffusing capacity [9]. Therefore, feelings of dyspnoea could in part be explained by early emphysematous changes [13]. However, we found that spirometric SAO was associated with symptoms independently of cigarette smoking. It has also been shown that FEF_25-75_ is a sensitive predictor of airways hypersensitivity in asthma [25], so it is plausible that transient exposure to allergic and non-allergic triggers may result in acute and short-lived bouts of respiratory symptoms. While we have access to pre- and post-bronchodilator measurements, we did not investigate this mechanism further as bronchodilator reversibility does not always differentiate between asthma and COPD in population-based studies [26].

### Cardiometabolic diseases

In terms of lung function, the FVC appears to have the strongest association with CVD [22]. The association of airflow obstruction with CVD is more ambiguous [27], while for measures of spirometric SAO, evidence is lacking. In this study, spirometric SAO, defined either using FEF_25-75_ or FEV_3_/FVC, was associated with CVD but not hypertension or diabetes. Isolated SAO was also associated with CVD for both parameters.

The association was slightly stronger for FEF_25-75_ than for the FEV_3_/FVC, which despite adjustment in our multivariate models, could be explained by the correlation between the FEF_25-75_ and FVC. However, when we excluded participants with low FVC, the magnitude of the association between spirometric SAO based on FEF_25-75_ and CVD did not materially change, making this explanation less likely. To rule out residual confounding by smoking from the association between CVD and spirometric SAO, we restricted our analysis to never smokers. This dismissed the association of CVD with spirometric SAO based on FEV_3_/FVC but not with spirometric SAO based on FEF_25-75_. Our finding that people with isolated spirometric SAO, i.e. in the absence of airflow obstruction, are more likely to have a diagnosis of CVD is interesting. A potential explanation for this is that spirometric SAO upregulates inflammatory processes. Castonzo et al. [28], showed that people with a lower FEF_25-75_ percent predicted had higher levels of C-reactive protein (CRP), a marker of systemic inflammation associated with increased risk of both heart disease and stroke [29]. However, it is also plausible that reverse causation plays a role, as it has been shown in mice that heart failure causes pulmonary remodelling, oedema, and fibrosis, all of which can impair lung function [30].

We found conflicting associations of hypertension with spirometric SAO, with no significant association for FEV_3_/FVC, and FEF_25-75_ associated with increased odds of hypertension in males and those with isolated spirometric SAO only. Few studies have investigated the association between spirometric SAO and hypertension. In a hospital-based study, Birhan et al. [31] compared the spirometry results of 61 hypertensive and 61 normotensive individuals. They found that hypertensive individuals had a significantly lower FEF_25-75_ compared to normotensive individuals. However, this finding was not adjusted for potential confounders, such as FVC [22].

Like hypertension, we found conflicting associations between spirometric SAO and diabetes. Spirometric SAO defined using FEV_3_/FVC was not associated with diabetes, whereas spirometric SAO using FEF_25-75_ was associated with reduced odds of diabetes. A South Korean study of over 17,000 healthy adults found no association between baseline spirometric SAO and risk of diabetes after 6 years [32]. These results are likely more applicable than those of the present study. Firstly, because it is a longitudinal study, better for investigating causality, and secondly, because the HbA1c blood test was used to diagnose diabetes.

The lack of association with two major risk factors for CVD, despite the association of spirometric SAO with CVD in this study, suggests at least two explanations. Either: (1) the mechanism by which spirometric SAO increases the risk of CVD does not act through pathways that increase blood pressure or impair blood glucose regulation, or (2) the association between spirometric SAO and CVD is not real and is confounded by some factor that we were unable to account for.

### Quality of life

We found that people with spirometric SAO are more likely to have worse QoL. However, we found no evidence of association of QoL with isolated spirometric SAO. These findings are plausible, as airflow limitation may not be severe enough to impact daily living. Contrary to our results, Dilkektasli et al. [11] reported that isolated spirometric SAO associated with lower QoL. However, their study population was restricted to former and current smokers, who have been shown to have a lower QoL than non-smokers [33]. We also found that the significant association between spirometric SAO and reduced QoL was mainly seen in males and not females. In the context of our results this makes sense, as males with spirometric SAO reported more respiratory symptoms, which have been shown to be independently associated with QoL [34].

## Strengths and limitations

Our study has several strengths. First, its large sample size and population-based design makes the results transferable to general populations. Spirometry was conducted by trained and certified technicians using the same protocol and model of spirometer, and lung function data was quality assured centrally with each curve visually inspected. A further strength is the administration of standardised questionnaires, in local languages, across study sites. Our study also has some limitations. The cross-sectional nature of the study precludes assessment of causality. In addition, there were instances of moderate heterogeneity in the association of spirometric SAO with symptoms and QoL. Therefore, caution should be taken when relating our pooled estimates to specific countries or world regions. Further limitations include the lack of a gold standard measure of spirometric SAO, as well as limited reference equations in suitable populations the FEV_3_/FVC ratio. This restricted our ability to use multi-ethnic reference values, which could have impacted the estimation of prevalence at some sites. However, the NHANES equations have been shown to give similar prevalence estimates for airflow obstruction regardless of race-correction [35], while recent evidence suggests that race-correction may misclassify individuals with underlying disease [36].

## Conclusions

The main novelty of our study concerns isolated spirometric SAO. Quanjer et al. [37] recommended against the use of FEF_25-75_ in the clinical setting as they found little evidence of isolated spirometric SAO in a sample of people with chronic respiratory disease. In contrast with their study, we have found that isolated spirometric SAO is common in general populations and is associated with respiratory symptoms. In addition, we have shown that isolated spirometric SAO has the potential to be used to detect people at risk of cardiovascular disease. Therefore, consideration should be given to the measurement of FEF_25-75_ and FEV_3_/FVC in clinical and general populations. Future research should aim to corroborate our findings and investigate whether those with isolated spirometric SAO go on to develop airflow obstruction or cardiovascular disease later in life.

## Supplementary Information

**Additional file 1. Table S1**. Pooled estimates for the association between spirometric small airways obstruction and respiratory symptoms in the BOLD study.** Table S2**. Pooled estimates for the association between spirometric small airways obstruction and cardiometabolic diseases in the BOLD study.** Table S3**. Pooled estimates for the association between spirometric small airways obstruction and physical and mental scores of quality of life (QoL) in the BOLD study.** Table S4**. Pooled estimates for the association of spirometric small airways obstruction with respiratory symptoms, cardiometabolic diseases and quality of life in never smokers from the BOLD study.** Table S5**. Pooled estimates for the association between small airways obstruction and cardiometabolic diseases among participants with normal FVC in the BOLD study.** Table S6 and S7**. Pooled estimates for the association of FEV_3_/FVC (%) and FEF_25–75_ (L/s) with respiratory symptoms and cardiometabolic diseases in all participants and those with a normal FEV_1_/FVC ratio.

## Data Availability

De-identified participant data and questionnaires may be shared, after publication, on a collaborative basis upon reasonable request made to Dr Amaral (a.amaral@imperial.ac.uk). Requesting researchers will be required to submit an analysis plan.

## Abbreviations

BOLD study: Burden of obstructive lung disease study
COPD: Chronic obstructive pulmonary disease
CAO: Chronic airflow obstruction
SAO: Small airways obstruction
FEF_25-75_: Mean forced expiratory flow rate between 25 and 75% of the FVC
FEV_1_: Forced expiratory volume in 1 s
FVC: Forced vital capacity
FEV_3_: Forced expiratory volume in 3 s
FEV_1_/FVC: Forced expiratory volume in 1 s as a ratio of the forced vital capacity
FEV_3_/FVC: Forced expiratory volume in 3 s as a ratio of the forced vital capacity
LLN: Lower limit of normal
FET_25-75_: Forced expiratory time between 25 and 75% of the forced vital capacity
CI: Confidence interval
SD: Standard deviation
WHO: World Health Organisation
CT: Computed tomography
Pre-BD: Pre bronchodilator
Post-BD: Post bronchodilator
BMI: Body mass index
ATS: American thoracic society
QoL: Quality of life
CVD: Cardiovascular disease
mMRC: Medical research council questionnaire

## References

[1] Knox-Brown B, Mulhern O, Feary J (2022). Spirometry parameters used to define small airways obstruction in population-based studies: systematic review. Respir Res.

[2] Trinkmann F, Watz H, Herth FJF (2020). Why do we still cling to spirometry for assessing small airway function?. Eur Respir J.

[3] Weibel ER (1963). Morphometry of the human lung.

[4] Kraft M, Richardson M, Hallmark B (2022). The role of small airway dysfunction in asthma control and exacerbations: a longitudinal, observational analysis using data from the ATLANTIS study. Lancet Respir Med.

[5] Crisafulli E, Pisi R, Aiello M (2017). Prevalence of small-airway dysfunction among COPD patients with different GOLD stages and its role in the impact of disease. Respiration.

[6] Schneider C, Bothner U, Jick SS (2010). Chronic obstructive pulmonary disease and the risk of cardiovascular diseases. Eur J Epidemiol.

[7] Xiao D, Chen Z, Wu S (2020). Prevalence and risk factors of small airway dysfunction, and association with smoking, in China: findings from a national cross-sectional study. Lancet Respir Med.

[8] Knox-Brown B, Patel J, Potts J (2023). Small airways obstruction and its risk factors in the Burden of Obstructive Lung Disease (BOLD) study: a multinational cross-sectional study. Lancet Glob Health.

[9] Morris ZQ, Coz A, Starosta D (2013). An isolated reduction of the FEV3/FVC ratio is an indicator of mild lung injury. Chest.

[10] Qin S, Yu X, Ma Q (2021). Quantitative CT analysis of small airway remodeling in patients with chronic obstructive pulmonary disease by a new image post-processing system. Int J Chron Obstruct Pulmon Dis.

[11] Dilektasli AG, Porszasz J, Casaburi R (2016). A novel spirometric measure identifies mild COPD unidentified by standard criteria. Chest.

[12] Ronish BE, Couper DJ, Barjaktarevic IZ (2022). Forced expiratory flow at 25%-75% Links COPD physiology to emphysema and disease severity in the SPIROMICS Cohort. Chronic Obstr Pulm Dis.

[13] Yee N, Markovic D, Buhr RG (2021). Significance of FEV(3)/FEV(6) in recognition of early airway disease in smokers at risk of development of COPD: analysis of the SPIROMICS cohort. Chest.

[14] Buist AS, Vollmer WM, Sullivan SD (2005). The Burden of Obstructive Lung Disease Initiative (BOLD): rationale and design. COPD.

[15] American Thoracic Society (1995). Standardization of Spirometry, 1994 Update. Am J Respir Crit Care Med.

[16] Hankinson JL, Odencrantz JR, Fedan KB (1999). Spirometric reference values from a sample of the general U.S. population. Am J Respir Crit Care Med.

[17] Hansen JE, Sun XG, Wasserman K (2006). Discriminating measures and normal values for expiratory obstruction. Chest.

[18] Gandek B, Ware JE, Aaronson NK (1998). Cross-validation of item selection and scoring for the SF-12 Health Survey in nine countries: results from the IQOLA. Project International Quality of Life Assessment. J Clin Epidemiol.

[19] Grønseth R, Vollmer WM, Hardie JA (2014). Predictors of dyspnoea prevalence: results from the BOLD study. Eur Respir J.

[20] Jousilahti P, Vartiainen E, Tuomilehto J (1999). Sex, age, cardiovascular risk factors, and coronary heart disease: a prospective follow-up study of 14 786 middle-aged men and women in Finland. Circulation.

[21] Wickrama KA, O'Neal CW, Lott RE (2012). Early community contexts, race/ethnicity and young adult CVD risk factors: the protective role of education. J Community Health.

[22] Kulbacka-Ortiz K, Triest FJJ, Franssen FME (2022). Restricted spirometry and cardiometabolic comorbidities: results from the international population based BOLD study. Respir Res.

[23] Hunter JE, Schmidt FL (2000). Fixed effects vs. random effects meta-analysis models: implications for cumulative research knowledge. Int J Select Assess.

[24] Mejza F, Gnatiuc L, Buist AS (2017). Prevalence and burden of chronic bronchitis symptoms: results from the BOLD study. Eur Respir J.

[25] Kim Y, Lee H, Chung SJ (2021). The usefulness of FEF(25–75) in predicting airway hyperresponsiveness to Mannitol. J Asthma Allergy.

[26] Janson C, Malinovschi A, Amaral AFS (2019). Bronchodilator reversibility in asthma and COPD: findings from three large population studies. Eur Respir J.

[27] Triest FJJ, Studnicka M, Franssen FME (2019). Airflow obstruction and cardio-metabolic comorbidities. COPD.

[28] Costanzo S, Magnacca S, Bonaccio M (2021). Reduced pulmonary function, low-grade inflammation and increased risk of total and cardiovascular mortality in a general adult population: prospective results from the Moli-sani study. Respir Med.

[29] Kaptoge S, Di Angelantonio E, Lowe G (2010). C-reactive protein concentration and risk of coronary heart disease, stroke, and mortality: an individual participant meta-analysis. Lancet.

[30] Chen Y, Guo H, Xu D (2012). Left ventricular failure produces profound lung remodeling and pulmonary hypertension in mice: heart failure causes severe lung disease. Hypertension.

[31] Birhan MM, Abebe Y (2018). Pulmonary function tests in hypertensive patients attending Zewditu Memorial Hospital, Addis Ababa, Ethiopia. Int J Hypertens.

[32] Lee HY, Shin J, Kim H (2021). Association between lung function and new-onset diabetes mellitus in healthy individuals after a 6-year follow-up. Endocrinol Metab (Seoul).

[33] García-Quero C, Carreras J, Martínez-Cerón E (2020). Small airway dysfunction impairs quality of life among smokers with no airflow limitation. Arch Bronconeumol (Engl Ed).

[34] Janson C, Marks G, Buist S (2013). The impact of COPD on health status: findings from the BOLD study. Eur Respir J.

[35] Tilert T, Dillon C, Paulose-Ram R (2013). Estimating the US prevalence of chronic obstructive pulmonary disease using pre- and post-bronchodilator spirometry: the National Health and Nutrition Examination Survey (NHANES) 2007–2010. Respir Res.

[36] Ekström M, Mannino D (2022). Research race-specific reference values and lung function impairment, breathlessness and prognosis: analysis of NHANES 2007–2012. Respir Res.

[37] Quanjer PH, Weiner DJ, Pretto JJ (2014). Measurement of FEF25-75% and FEF75% does not contribute to clinical decision making. Eur Respir J.

