# Supplementary material for: The association of spirometric small airways obstruction with respiratory symptoms, cardiometabolic diseases, and quality of life: results from the Burden of Obstructive Lung Disease (BOLD) study
Source: Respir Res. 2023 May 23;24:137. doi: 10.1186/s12931-023-02450-1 (PMC10207810; doi:10.1186/s12931-023-02450-1)
Supplement: Supplementary file 1 — Additional file 1. Table S1. Pooled estimates for the association between spirometric small airways obstruction and respiratory symptoms in the BOLD study. Table S2. Pooled estimates for the association between spirometric small airways obstruction and cardiometabolic diseases in the BOLD study. Table S3. Pooled estimates for the association between spirometric small airways obstruction and physical and mental scores of quality of life (QoL) in the BOLD study. Table S4. Pooled estimates for the association of spirometric small airways obstruction with respiratory symptoms, cardiometabolic diseases and quality of life in never smokers from the BOLD study. Table S5. Pooled estimates for the association between small airways obstruction and cardiometabolic diseases among participants with normal FVC in the BOLD study. Table S6 and S7. Pooled estimates for the association of FEV3/FVC (%) and FEF25–75 (L/s) with respiratory symptoms and cardiometabolic diseases in all participants and those with a normal FEV1/FVC ratio. [file 12931_2023_2450_MOESM1_ESM.docx]

**Additional file**

**Title:** The association of spirometric small airways obstruction with cardiometabolic disease, respiratory symptoms, and quality of life: Results from the Burden of Obstructive Lung Disease (BOLD) study.

**Authors:** Ben Knox-Brown^1^, Jaymini Patel^1^, James Potts^1^, Rana Ahmed^2^, Althea Aquart-Stewart^3^, Cristina Barbara^4,5^, A Sonia Buist^6^, Hamid Hacene Cherkaski^7^, Meriam Denguezli^8^, Mohammed Elbiaze^9^, Gregory E. Erhabor^10^, Frits M.E. Franssen^11,12^, Mohammed Al Ghobain^13^, Thorarinn Gislason^14,15^, Christer Janson^16^, Ali Kocabaş^17^, David Mannino^18,19^, Guy Marks^20,21,22^, Kevin Mortimer^23,24^, Asaad Ahmed Nafees^25^, Daniel Obaseki^26^, Stefanni Nonna M. Paraguas^27,28^, Li Cher Loh^29^, Abdul Rashid^29^, Sundeep Salvi^30,31^, Terence Seemungal^32^, Michael Studnicka^33^, Wan C Tan^34^, Emiel F.M. Wouters^35,36^, Hazim Abozid^36^, Alexander Mueller^36^, Peter Burney^1^, Andre F.S. Amaral^1^, for the BOLD Collaborative Research Group.

**Contents**

**Page 2…………** Table S1. Pooled estimates for the association between spirometric small airways obstruction and respiratory symptoms in the BOLD study.

**Page 3…………** Table S2. Pooled estimates for the association between spirometric small airways obstruction and cardiometabolic diseases in the BOLD study.

**Page 4…………** Table S3. Pooled estimates for the association between spirometric small airways obstruction and physical and mental scores of quality of life (QoL) in the BOLD study.

**Page 5…………** Table S4. Pooled estimates for the association of spirometric small airways obstruction with respiratory symptoms, cardiometabolic diseases and quality of life in never smokers from the BOLD study.

**Page 6…………** Table S5. Pooled estimates for the association between small airways obstruction and cardiometabolic diseases among participants with normal FVC in the BOLD study.

**Page 7…………** Table S6 and S7. Pooled estimates for the association of FEV_3_/FVC (%) and FEF_25-75_ (L/s) with respiratory symptoms and cardiometabolic diseases in all participants and those with a normal FEV_1_/FVC ratio.

**Table S1. Pooled estimates for the association between spirometric small airways obstruction and respiratory symptoms in the BOLD study.**

|  | **SAO FEV_3_/FVC** | | | | | | **SAO FEF_25-75_** | | | | | |
| --- | --- | --- | --- | --- | --- | --- | --- | --- | --- | --- | --- | --- |
|  | **Unadjusted** | | | **Adjusted** | | | **Unadjusted** | | | **Adjusted** | | |
|  | **OR (95% CI)** | **Heterogeneity** | | **OR (95% CI)** | **Heterogeneity** | | **OR (95% CI)** | **Heterogeneity** | | **OR (95% CI)** | **Heterogeneity** | |
|  |  | **I^2^ %** | **p-value** |  | **I^2^ %** | **p-value** |  | **I^2^ %** | **p-value** |  | **I^2^ %** | **p-value** |
| **Dyspnoea** |  |  |  |  |  |  |  |  |  |  |  |  |
| SAO | 2.29 (1.93, 2.72) | 50.3 | <0.001 | 2.12 (1.66, 2.71) | 54.2 | 0.001 | 2.53 (2.15, 2.97) | 50.7 | <0.001 | 2.16 (1.77, 2.70) | 51.5 | 0.001 |
| SAO - Male | 3.59 (2.81, 4.58) | 43.1 | 0.005 | 2.95 (1.95, 4.46) | 51.3 | 0.001 | 3.56 (2.78, 4.60) | 47.5 | <0.001 | 3.56 (2.39, 5.31) | 56.3 | <0.001 |
| SAO - Female | 1.91 (1.50, 2.44) | 42.4 | 0.003 | 1.72 (1.20, 2.45) | 58.2 | <0.001 | 1.91 (1.60, 2.29) | 31.4 | 0.014 | 1.98 (1.57, 2.50) | 36.3 | 0.004 |
| Isolated SAO | 1.08 (0.81, 1.44) | 30.4 | 0.044 | 1.10 (0.75, 1.62) | 42.3 | 0.014 | 2.09 (1.64, 2.67) | 46.5 | <0.001 | 1.45 (1.13, 1.86) | 23.6 | 0.061 |
| **Chronic cough** |  |  |  |  |  |  |  |  |  |  |  |  |
| SAO | 2.45 (2.06, 2.91) | 25.4 | 0.065 | 1.97 (1.57, 2.47) | 28.9 | 0.080 | 2.70 (2.32, 3.15) | 12.0 | 0.308 | 2.56 (2.08, 3.15) | 15.5 | 0.217 |
| SAO - Male | 3.03 (2.42, 3.80) | 15.5 | 0.342 | 2.60 (1.89, 3.59) | 3.9 | 0.467 | 2.95 (2.38, 3.65) | 14.9 | 0.158 | 2.51 (1.86, 3.92) | 13.0 | 0.152 |
| SAO - Female | 2.19 (1.76, 2.73) | 11.4 | 0.253 | 1.85 (1.35, 2.54) | 0.0 | 0.960 | 2.52 (2.00, 3.17) | 29.2 | 0.087 | 2.45 (1.87, 3.19) | 0.0 | 0.540 |
| Isolated SAO | 1.75 (1.32, 2.32) | 7.9 | 0.689 | 1.27 (0.82, 1.99) | 23.9 | 0.310 | 2.19 (1.71, 2.80) | 22.4 | 0.363 | 2.01 (1.54, 2.63) | 0.0 | 0.794 |
| **Chronic phlegm** |  |  |  |  |  |  |  |  |  |  |  |  |
| SAO | 2.85 (2.21, 3.66) | 62.3 | <0.001 | 2.08 (1.61, 2.68) | 41.1 | 0.004 | 2.59 (2.13, 3.15) | 38.1 | 0.005 | 2.29 (1.77, 4.05) | 45.0 | 0.002 |
| SAO - Male | 2.70 (2.00, 3.64) | 52.5 | <0.001 | 2.17 (1.57, 3.01) | 16.4 | 0.191 | 3.32 (2.83, 3.91) | 16.0 | 0.299 | 3.23 (2.52, 4.13) | 23.9 | 0.093 |
| SAO - Female | 2.71 (2.05, 3.60) | 28.5 | 0.139 | 1.92 (1.41, 2.62) | 0.0 | 0.811 | 2.56 (1.94, 3.38) | 41.9 | 0.009 | 2.34 (1.70, 3.22) | 20.2 | 0.119 |
| Isolated SAO | 1.67 (1.23, 2.29) | 21.3 | 0.332 | 0.97 (0.67, 1.40) | 0.0 | 0.966 | 2.12 (1.63, 2.76) | 13.0 | 0.450 | 2.15 (1.55, 2.98) | 29.7 | 0.059 |
| **Wheeze** |  |  |  |  |  |  |  |  |  |  |  |  |
| SAO | 2.60 (2.21, 3.04) | 57.9 | <0.001 | 2.57 (2.08, 3.19) | 62.2 | <0.001 | 3.08 (2.73, 3.46) | 21.4 | 0.179 | 2.87 (2.5, 3.4) | 27.2 | 0.145 |
| SAO - Male | 2.47 (2.05, 3.00) | 39.1 | 0.021 | 2.71 (2.03, 3.60) | 51.7 | 0.002 | 4.24 (3.42, 5.26) | 26.3 | 0.074 | 3.16 (2.29, 4.65) | 29.0 | 0.227 |
| SAO - Female | 2.60 (2.18, 3.09) | 14.6 | 0.419 | 2.50 (2.04, 3.17) | 6.8 | 0.378 | 2.74 (2.39, 3.15) | 0.0 | 0.679 | 2.91 (2.42, 3.51) | 0.0 | 0.646 |
| Isolated SAO | 1.31 (1.08, 1.59) | 10.8 | 0.596 | 1.28 (1.01, 1.62) | 13.0 | 0.500 | 2.13 (1.83, 2.0) | 0.0 | 0.728 | 1.93 (1.59, 2.33) | 0.0 | 0.308 |

*SAO: Small airways obstruction – FEF_25-75_ or FEV_3_/FVC less than the lower limit of normal (LLN). SAO – male/female: stratified by sex. Isolated SAO: FEF_25-75_ or FEV_3_/FVC less than the lower limit of normal with FEV_1_/FVC ≥LLN. Dyspnoea measured according to mMRC Dyspnoea scale: 0-1= minimal/no breathlessness, ≥2= significant breathlessness. Chronic cough: cough on most days for 3 months each year. Chronic Phlegm: Phlegm on most days three months each year. Wheeze: Wheezing or whistling in the chest at any time in the last 12 months. I^2^ values of 0%, 25%, 50%, and 75% considered no, low, moderate, and high heterogeneity. P-value represents significance for Chi-squared test for heterogeneity of pooled estimates, p<0.05= significant. Covariates in the adjusted model: sex, education level, body mass index, smoking status, smoking pack-years,* *passive smoking, occupational exposure to dust, use of solid fuels for cooking/heating for >6 months in a lifetime, reported doctor-diagnosed or history of tuberculosis, spirometric restriction family history of COPD, and for Dyspnoea addition of CVD. Estimates based on the analysis of 28 sites. The following sites could not be included in the analysis either due to a low number of participants reporting respiratory symptoms or singularity in the data: Benin (Sémé-Kpodji), Norway (Bergen), Malawi (Blantyre), China (Guangzhou), Germany (Hannover), Cameroon (Limbe), India (Mumbai) (Mysore), Austria (Salzburg), Tunisia (Sousse), Australia (Sydney), Albania (Tirana), Sweden (Uppsala).*

**Table S2. Pooled estimates for the association between spirometric small airways obstruction and cardiometabolic diseases in the BOLD study.**

|  | **Pre-bronchodilator SAO FEV_3_/FVC** | | | | | | **Pre-bronchodilator SAO FEF_25-75_** | | | | | |
| --- | --- | --- | --- | --- | --- | --- | --- | --- | --- | --- | --- | --- |
|  | **Unadjusted** | | | **Adjusted** | | | **Unadjusted** | | | **Adjusted** | | |
|  | **OR (95% CI)** | **Heterogeneity** | | **OR (95% CI)** | **Heterogeneity** | | **OR (95% CI)** | **Heterogeneity** | | **OR (95% CI)** | **Heterogeneity** | |
|  |  | **I^2^ %** | **p-value** |  | **I^2^ %** | **p-value** |  | **I^2^ %** | **p-value** |  | **I^2^ %** | **p-value** |
| **Cardiovascular disease** |  |  |  |  |  |  |  |  |  |  |  |  |
| SAO | 1.48 (1.24, 1.75) | 48.4 | 0.004 | 1.23 (1.02, 1.49) | 40.1 | 0.034 | 1.21 (1.06, 1.39) | 8.2 | 0.456 | 1.30 (1.11, 1.52) | 0.0 | 0.445 |
| SAO - Male | 1.79 (1.51, 2.11) | 0.0 | 0.733 | 1.22 (0.98, 1.52) | 0.0 | 0.845 | 1.40 (1.14, 1.70) | 0.0 | 0.385 | 1.26 (0.96, 1.65) | 1.3 | 0.473 |
| SAO - Female | 1.56 (1.23, 1.99) | 48.3 | 0.003 | 1.35 (0.99, 1.84) | 49.9 | 0.009 | 1.25 (1.06, 1.47) | 4.7 | 0.467 | 1.43 (1.14, 1.79) | 0.0 | 0.512 |
| Isolated SAO | 1.43 (1.18, 1.75) | 0.0 | 0.829 | 1.33 (1.06, 1.67) | 2.6 | 0.539 | 1.34 (1.09, 1.65) | 0.0 | 0.937 | 1.46 (1.10, 1.89) | 5.7 | 0.482 |
| **Hypertension** |  |  |  |  |  |  |  |  |  |  |  |  |
| SAO | 1.14 (1.00, 1.30) | 59.9 | <0.001 | 1.05 (0.94, 1.18) | 23.6 | 0.083 | 1.05 (0.95, 1.15) | 13.1 | 0.386 | 1.07 (0.96, 1.20) | 16.1 | 0.460 |
| SAO - Male | 1.38 (1.17, 1.62) | 39.3 | 0.011 | 1.10 (0.97, 1.27) | 0.0 | 0.531 | 1.21 (1.07, 1.39) | 0.0 | 0.932 | 1.26 (1.09. 1.47) | 0.0 | 0.888 |
| SAO - Female | 1.09 (0.92, 1.28) | 43.5 | 0.005 | 0.98 (0.80, 1.19) | 47.0 | 0.001 | 0.93 (0.83, 1.05) | 8.0 | 0.514 | 1.04 (0.91, 1.21) | 12.4 | 0.383 |
| Isolated SAO | 1.02 (0.88, 1.19) | 2.3 | 0.386 | 1.06 (0.89, 1.26) | 12.0 | 0.100 | 1.13 (1.00, 1.28) | 3.3 | 0.663 | 1.19 (1.03, 1.40) | 5.0 | 0.498 |
| **Diabetes** |  |  |  |  |  |  |  |  |  |  |  |  |
| SAO | 1.06 (0.89, 1.28) | 29.0 | 0.202 | 0.86 (0.72, 1.03) | 0.0 | 0.893 | 0.90 (0.77, 1.06) | 15.0 | 0.414 | 0.75 (0.63, 0.90) | 5.5 | 0.285 |
| SAO - Male | 1.20 (0.96, 1.52) | 22.6 | 0.322 | 0.80 (0.62, 1.04) | 0.0 | 0.875 | 1.11 (0.91, 1.36) | 0.0 | 0.958 | 0.79 (0.61, 1.00) | 0.0 | 0.645 |
| SAO - Female | 1.04 (0.84, 1.29) | 1.1 | 0.778 | 0.87 (0.67, 1.14) | 8.5 | 0.578 | 0.78 (0.64, 0.96) | 13.1 | 0.599 | 0.69 (0.56, 0.84) | 0.0 | 0.563 |
| Isolated SAO | 1.32 (1.01, 1.74) | 24.2 | 0.193 | 1.05 (0.80, 1.37) | 0.0 | 0.444 | 1.08 (0.83, 1.41) | 38.7 | 0.028 | 1.01 (0.74, 1.38) | 40.5 | 0.020 |

*SAO: Small airways obstruction – FEF_25-75_ or FEV_3_/FVC less than the lower limit of normal (LLN). SAO – male/female: stratified by sex. SAO – excluding restriction: excluding those with a FVC <LLN. Isolated SAO: FEF_25-75_ or FEV_3_/FVC less than the lower limit of normal with FEV_1_/FVC ≥LLN. Cardiovascular disease: self-reported history of heart disease or stroke. I^2^ values of 0%, 25%, 50%, and 75% considered no, low, moderate, and high heterogeneity. P-value represents significance for Chi-squared test for heterogeneity of pooled estimates, p<0.05= significant. Covariates in the adjusted model: sex, education level, body mass index, smoking status, smoking pack-years and spirometric restriction. The following sites could not be included in the analysis either due to a low number of participants reporting co-morbidity or singularity in the data: For CVD; Malawi (Blantyre), Malawi (Chikwawa), Nigeria (Ife), Cameroon (Limbe), India (Mysore), India (Srinagar), Malaysia (Penang), Sudan (Gezeira), Morocco (Fes), China (Guangzhou), Jamaica (Kingston), Trinidad & Tobago (Port of Spain), Saudi Arabia (Riyadh), and Albania (Tirana). For Hypertension; Benin (Sémé-Kpodji), Malawi (Chikwawa), and Sudan (Gezeira). For diabetes; India (Pune), Malawi (Chikwawa), Morocco (Fes), China (Guangzhou), Nigeria (Ife), Kyrgyzstan (Naryn), Cameroon (Limbe), Philippines (Manilla), India (Mumbai), Malaysia (Penang).*

**Table S3.** **Pooled estimates for the association between spirometric small airways obstruction and physical and mental scores of quality of life (QoL) in the BOLD study.**

|  | **SAO FEV_3_/FVC** | | | | | | **SAO FEF_25-75_** | | | | | |
| --- | --- | --- | --- | --- | --- | --- | --- | --- | --- | --- | --- | --- |
|  | **Unadjusted** | | | **Adjusted** | | | **Unadjusted** | | | **Adjusted** | | |
|  | **Regression coefficient**  **(95% CI)** | **Heterogeneity** | | **Regression coefficient**  **(95% CI)** | **Heterogeneity** | | **Regression coefficient**  **(95% CI)** | **Heterogeneity** | | **Regression coefficient**  **(95% CI)** | **Heterogeneity** | |
|  |  | **I^2^ %** | **p-value** |  | **I^2^ %** | **p-value** |  | **I^2^ %** | **p-value** |  | **I^2^ %** | **p-value** |
| **QoL Physical** |  |  |  |  |  |  |  |  |  |  |  |  |
| SAO | -2.10 (-2.75, -1.45) | 71.6 | <0.001 | -1.16 (-1.72, -0.61) | 61.1 | <0.001 | -1.78 (-2.32, -1.23) | 65.3 | <0.001 | -1.18 (-1.64, -0.72) | 54.1 | 0.001 |
| SAO - Male | -2.85 (-3.65, -2.06) | 72.3 | <0.001 | -1.53 (-2.27, -0.78) | 65.4 | <0.001 | -2.50 (-3.35, -1.67) | 71.3 | <0.001 | -1.80 (-2.61, -0.98) | 68.6 | <0.001 |
| SAO - Female | -1.14 (-1.84, -0.45) | 46.2 | 0.002 | -0.70 (-1.57, 0.19) | 69.7 | <0.001 | -0.61 (-1.09, -0.12) | 33.5 | 0.026 | -0.55 (-0.97, -0.14) | 27.6 | 0.111 |
| Isolated SAO | 0.20 (-0.63, 1.04) | 69.4 | <0.001 | 0.03 (-0.73, 0.80) | 58.5 | <0.001 | -0.92 (-1.70, -0.15) | 70.6 | <0.001 | -0.69 (-1.35, -0.02) | 76.2 | <0.001 |
| **QoL Mental** |  |  |  |  |  |  |  |  |  |  |  |  |
| SAO | -0.81 (-1.36, -0.25) | 52.3 | <0.001 | -0.79 (-1.34, -0.24) | 41.4 | 0.013 | -1.05 (-1.41, -0.69) | 12.1 | 0.291 | -0.76 (-1.19, -0.33) | 28.3 | 0.200 |
| SAO - Male | -0.98 (-1.57, -0.41) | 33.6 | 0.042 | -0.78 (-1.35, -0.23) | 14.4 | 0.261 | -1.06 (-1.48, -0.65) | 0.0 | 0.609 | -0.87 (-1.51, -0.24) | 37.4 | 0.040 |
| SAO - Female | -0.86 (-1.65, -0.08) | 49.8 | <0.001 | -0.63 (-1.61, 0.36) | 62.5 | <0.001 | -0.75 (-1.29, -0.21) | 24.8 | 0.053 | -0.43 (-1.00, 0.14) | 29.6 | 0.054 |
| Isolated SAO | -0.28 (-1.10, 0.53) | 59.1 | <0.001 | 1.08 (-0.07, 2.24) | 74.7 | <0.001 | -0.70 (-1.26, -0.12) | 21.2 | 0.139 | 0.07 (-0.56, 0.70) | 25.4 | 0.158 |

*SAO: Small airways obstruction – FEF_25-75_ or FEV_3_/FVC less than the lower limit of normal (LLN). SAO – male/female: stratified by sex. Isolated SAO: FEF_25-75_ or FEV_3_/FVC less than the lower limit of normal with FEV_1_/FVC ≥LLNPhysical and mental QoL measured using the SF-12 questionnaire. Negative regression coefficient indicates that having SAO is associated with a reduction in SF-12 score in comparison to not having SAO. I^2^ values of 0%, 25%, 50%, and 75% considered no, low, moderate, and high heterogeneity. P-value represents significance for Chi-squared test for heterogeneity of pooled estimates, p<0.05= significant. Covariates in the adjusted model: sex, education level, body mass index, smoking status, smoking pack-years, passive smoking, occupational exposure to dust, use of solid fuels for cooking/heating for >6 months in a lifetime, reported doctor-diagnosed or history of tuberculosis, spirometric restriction family history of COPD, CVD, hypertension, and diabetes. Estimates based on the analysis of 31 sites, the following sites could not be included in the analysis either due to low response rate to the questionnaire; Turkey (Adana) and China (Guangzhou) or where QoL was measured using a different tool; Benin (Sémé-Kpodji), Cameroon (Limbe), Jamaica (Kingston), Kyrgyzstan (Chui), Kyrgyzstan (Naryn), Malaysia (Penang), Pakistan (Karachi), Sri Lanka (Colombo).*

**Table S4. Pooled estimates for the association of spirometric small airways obstruction with respiratory symptoms, cardiometabolic diseases and quality of life in never smokers from the BOLD study.**

| **Never smokers (n=13,642)** | **Pre-bronchodilator SAO FEV_3_/FVC** | | | **Pre-bronchodilator SAO FEF_25-75_** | | |
| --- | --- | --- | --- | --- | --- | --- |
|  | **OR (95% CI)** | **Heterogeneity** | | **OR (95% CI)** | **Heterogeneity** | |
|  |  | **I^2^ %** | **p-value** |  | **I^2^ %** | **p-value** |
| **Symptoms** |  |  |  |  |  |  |
| Dyspnoea | 2.26 (1.60, 3.19) | 52.2 | 0.001 | 2.53 (1.88, 3.40) | 55.6 | <0.001 |
| Chronic cough | 2.12 (1.54, 2.93) | 27.9 | 0.084 | 2.58 (1.94, 3.60) | 18.0 | 0.087 |
| Chronic Phlegm | 2.74 (1.85, 4.07) | 31.7 | 0.050 | 3.22 (1.79, 5.80) | 79.3 | <0.001 |
| Wheeze | 3.29 (2.59, 4.19) | 20.5 | 0.109 | 3.37 (2.81, 4.04) | 0.0 | 0.153 |
| **Cardiometabolic disease** |  |  |  |  |  |  |
| Cardiovascular disease | 1.19 (0.98, 1.46) | 0.0 | 0.787 | 1.45 (1.15, 1.82) | 11.7 | 0.483 |
| Hypertension | 1.01 (0.85, 1.20) | 37.0 | 0.012 | 1.10 (0.97, 1.25) | 10.3 | 0.641 |
| Diabetes | 0.93 (0.74, 1.17) | 0.0 | 0.699 | 0.84 (0.69, 1.02) | 1.6 | 0.326 |
|  | **Regression coefficient**  **(95% CI)** | **I^2^ %** | **p-value** | **Regression coefficient**  **(95% CI)** | **I^2^ %** | **p-value** |
| **QoL** |  |  |  |  |  |  |
| Physical | -0.68 (-1.29, -0.10) | 50.8 | 0.003 | -0.84 (-1.38, -0.30) | 52.5 | <0.001 |
| Mental | -1.31 (-1.9, -0.7) | 31.5 | 0.059 | -0.79 (-1.21, -0.38) | 0.0 | 0.713 |

*SAO: Small airways obstruction – FEF_25-75_ or FEV_3_/FVC less than the lower limit of normal (LLN). Cardiovascular disease: self-reported history of heart disease or stroke. Covariates in the adjusted model: sex, education level, body mass index, smoking status, smoking pack-years and spirometric restriction. The following sites could not be included in the analysis either due to a low number of participants reporting co-morbidity or singularity in the data: For CVD; Malawi (Blantyre), Malawi (Chikwawa), Nigeria (Ife), Cameroon (Limbe), India (Mysore), India (Srinagar), Malaysia (Penang), Sudan (Gezeira), Morocco (Fes), China (Guangzhou), Jamaica (Kingston), Trinidad & Tobago (Port of Spain), Saudi Arabia (Riyadh), and Albania (Tirana). For Hypertension; Benin (Sémé-Kpodji), Malawi (Chikwawa), and Sudan (Gezeira). For diabetes; India (Pune), Malawi (Chikwawa), Morocco (Fes), China (Guangzhou), Nigeria (Ife), Kyrgyzstan (Naryn), Cameroon (Limbe), Philippines (Manilla), India (Mumbai), Malaysia (Penang). Dyspnoea measured according to mMRC Dyspnoea scale: 0-1= minimal/no breathlessness, ≥2= significant breathlessness. Chronic cough: cough on most days for 3 months each year. Chronic Phlegm: Phlegm on most days three months each year. Wheeze: Wheezing or whistling in the chest at any time in the last 12 months. Covariates in the adjusted model: sex, education level, body mass index, smoking status, smoking pack-years,* *passive smoking, occupational exposure to dust, use of solid fuels for cooking/heating for >6 months in a lifetime, reported doctor-diagnosed or history of tuberculosis, spirometric restriction family history of COPD, and for Dyspnoea addition of CVD. Estimates based on the analysis of 28 sites. The following sites could not be included in the analysis either due to a low number of participants reporting respiratory symptoms or singularity in the data: Benin (Sémé-Kpodji), Norway (Bergen), Malawi (Blantyre), China (Guangzhou), Germany (Hannover), Cameroon (Limbe), India (Mumbai) (Mysore), Austria (Salzburg), Tunisia (Sousse), Australia (Sydney), Albania (Tirana), Sweden (Uppsala). Physical and mental QoL measured using the SF-12 questionnaire. Negative regression coefficient indicates that having SAO is associated with a reduction in SF-12 score in comparison to not having SAO. Covariates in the adjusted model: As for symptoms with addition of CVD, hypertension, and diabetes. Estimates based on the analysis of 31 sites, the following sites could not be included in the analysis either due to low response rate to the questionnaire; Turkey (Adana) and China (Guangzhou) or where QoL was measured using a different tool; Benin (Sémé-Kpodji), Cameroon (Limbe), Jamaica (Kingston), Kyrgyzstan (Chui), Kyrgyzstan (Naryn), Malaysia (Penang), Pakistan (Karachi), Sri Lanka (Colombo). I^2^ values of 0%, 25%, 50%, and 75% considered no, low, moderate, and high heterogeneity. P-value represents significance for Chi-squared test for heterogeneity of pooled estimates, p<0.05= significant.*

**Table S5. Pooled estimates for the association between spirometric small airways obstruction and cardiometabolic diseases among participants with normal FVC in the BOLD study.**

|  | **Pre-bronchodilator SAO FEV_3_/FVC** | | | | | | **Pre-bronchodilator SAO FEF_25-75_** | | | | | |
| --- | --- | --- | --- | --- | --- | --- | --- | --- | --- | --- | --- | --- |
|  | **Unadjusted** | | | **Adjusted** | | | **Unadjusted** | | | **Adjusted** | | |
|  | **OR (95% CI)** | **Heterogeneity** | | **OR (95% CI)** | **Heterogeneity** | | **OR (95% CI)** | **Heterogeneity** | | **OR (95% CI)** | **Heterogeneity** | |
|  |  | **I^2^ %** | **p-value** |  | **I^2^ %** | **p-value** |  | **I^2^ %** | **p-value** |  | **I^2^ %** | **p-value** |
| **Cardiovascular disease** | 1.55 (1.26, 1.91) | 50.8 | <0.001 | 1.22 (0.98, 1.54) | 44.3 | 0.005 | 1.21 (0.99, 1.47) | 23.1 | 0.136 | 1.38 (1.13, 1.68) | 0.0 | 0.635 |
| **Hypertension** | 1.16 (0.96, 1.38) | 70.0 | <0.001 | 1.06 (0.88, 1.26) | 54.1 | <0.001 | 1.08 (0.93, 1.25) | 33.6 | 0.042 | 1.22 (1.08, 1.39) | 2.2 | 0.532 |
| **Diabetes** | 1.09 (0.91, 1.31) | 8.5 | 0.706 | 0.92 (0.74, 1.16) | 0.0 | 0.982 | 0.87 (0.71, 1.06) | 0.0 | 0.923 | 0.86 (0.67, 1.11) | 0.0 | 0.816 |

*SAO: Small airways obstruction – FEF_25-75_ or FEV_3_/FVC less than the lower limit of normal (LLN). SAO – male/female: stratified by sex. SAO – excluding restriction: excluding those with a FVC <LLN. Isolated SAO: FEF_25-75_ or FEV_3_/FVC less than the lower limit of normal with FEV_1_/FVC ≥LLN. Cardiovascular disease: self-reported history of heart disease or stroke. I^2^ values of 0%, 25%, 50%, and 75% considered no, low, moderate, and high heterogeneity. P-value represents significance for Chi-squared test for heterogeneity of pooled estimates, p<0.05= significant. Covariates in the adjusted model: sex, education level, body mass index, smoking status, smoking pack-years and spirometric restriction. The following sites could not be included in the analysis either due to a low number of participants reporting co-morbidity or singularity in the data: For CVD; Malawi (Blantyre), Malawi (Chikwawa), Nigeria (Ife), Cameroon (Limbe), India (Mysore), India (Srinagar), Malaysia (Penang), Sudan (Gezeira), Morocco (Fes), China (Guangzhou), Jamaica (Kingston), Trinidad & Tobago (Port of Spain), Saudi Arabia (Riyadh), and Albania (Tirana). For Hypertension; Benin (Sémé-Kpodji), Malawi (Chikwawa), and Sudan (Gezeira). For diabetes; India (Pune), Malawi (Chikwawa), Morocco (Fes), China (Guangzhou), Nigeria (Ife), Kyrgyzstan (Naryn), Cameroon (Limbe), Philippines (Manilla), India (Mumbai), Malaysia (Penang).*

|  | **FEV_3_/FVC (%)** | | | **FEF_25-75_ (L/s)** | | |
| --- | --- | --- | --- | --- | --- | --- |
|  | **OR (95% CI)** | **Heterogeneity** | | **OR (95% CI)** | **Heterogeneity** | |
|  |  | **I^2^ %** | **p-value** |  | **I^2^ %** | **p-value** |
| **Symptoms** |  |  |  |  |  |  |
| Dyspnoea | 0.94 (0.92, 0.97) | 61.7 | <0.0001 | 0.63 (0.52, 0.76) | 69.9 | <0.0001 |
| Chronic cough | 0.94 (0.91, 0.96) | 55.2 | <0.0001 | 0.61 (0.50, 0.74) | 34.4 | 0.011 |
| Chronic Phlegm | 0.93 (0.89, 0.96) | 62.3 | <0.0001 | 0.60 (0.45 (0.81) | 75.9 | <0.0001 |
| Wheeze | 0.91 (0.89, 0.92) | 25.4 | 0.058 | 0.49 (0.43, 0.56) | 31.2 | 0.007 |
| **Cardiometabolic disease** |  |  |  |  |  |  |
| Cardiovascular disease | 0.96 (0.95, 0.98) | 14.0 | 0.200 | 0.88 (0.80, 0.96) | 32.6 | 0.010 |
| Hypertension | 1.00 (0.99, 1.01) | 16.9 | 0.132 | 0.92 (0.87, 0.97) | 18.7 | 0.234 |
| Diabetes | 1.02 (1.00, 1.03) | 0.0 | 0.777 | 1.09 (0.99, 1.20) | 36.4 | 0.033 |

**Table S6. Pooled estimates for the association of FEV_3_/FVC (%) and FEF_25-75_ (L/s) with respiratory symptoms and cardiometabolic diseases**

*Result interpreted as per 1% increase in FEV_3_/FVC and 1 L/s increase in FEF_25-75._ FEV_3_/FVC: forced expiratory volume in three seconds as a ratio of the forced vital capacity; FEF_25-75_: Mean forced expiratory flow rate between 25% and 75% of the forced vital capacity. Cardiovascular disease: self-reported history of heart disease or stroke. Covariates in the adjusted model: sex, education level, body mass index, smoking status, smoking pack-years and spirometric restriction. Dyspnoea measured according to mMRC Dyspnoea scale: 0-1= minimal/no breathlessness, ≥2= significant breathlessness. Chronic cough: cough on most days for 3 months each year. Chronic Phlegm: Phlegm on most days three months each year. Wheeze: Wheezing or whistling in the chest at any time in the last 12 months. Covariates in the adjusted model: sex, education level, body mass index, smoking status, smoking pack-years, passive smoking, occupational exposure to dust, use of solid fuels for cooking/heating for >6 months in a lifetime, reported doctor-diagnosed or history of tuberculosis, spirometric restriction family history of COPD, and for Dyspnoea addition of CVD. I^2^ values of 0%, 25%, 50%, and 75% considered no, low, moderate, and high heterogeneity. P-value represents significance for Chi-squared test for heterogeneity of pooled estimates, p<0.05= significant.*

|  | **FEV_3_/FVC (%)** | | | **FEF_25-75_ (L/s)** | | |
| --- | --- | --- | --- | --- | --- | --- |
|  | **OR (95% CI)** | **Heterogeneity** | | **OR (95% CI)** | **Heterogeneity** | |
|  |  | **I^2^ %** | **p-value** |  | **I^2^ %** | **p-value** |
| **Symptoms** |  |  |  |  |  |  |
| Dyspnoea | 0.98 (0.96, 1.00) | 5.0 | 0.221 | 0.80 (0.72, 0.88) | 10.8 | 0.356 |
| Chronic cough | 0.99 (0.96, 1.02) | 17.8 | 0.083 | 0.80 (0.70, 0.92) | 0.8 | 0.381 |
| Chronic Phlegm | 1.00 (0.97, 1.02) | 0.0 | 0.323 | 0.85 (0.73, 0.99) | 29.2 | 0.035 |
| Wheeze | 0.96 (0.95, 0.98) | 3.9 | 0.692 | 0.67 (0.59, 0.76) | 44.9 | 0.005 |
| **Cardiometabolic disease** |  |  |  |  |  |  |
| Cardiovascular disease | 0.98 (0.96, 1.00) | 0.0 | 0.280 | 0.87 (0.78, 0.97) | 30.3 | 0.082 |
| Hypertension | 0.99 (0.98, 1.01) | 5.0 | 0.134 | 0.90 (0.51, 0.96) | 9.1 | 0.261 |
| Diabetes | 1.01 (0.99, 1.04) | 50.8 | <0.0001 | 1.05 (0.92, 1.21) | 55.7 | <0.0001 |

**Table S7. Pooled estimates for the association of FEV_3_/FVC (%) and FEF_25-75_ (L/s) with respiratory symptoms and cardiometabolic diseases in those with a normal FEV_1_/FVC ratio**

*Result interpreted as per 1% increase in FEV_3_/FVC and 1 L/s increase in FEF_25-75_. Normal FEV_1_/FVC ratio if result is ≥ the lower limit of normal. FEV_3_/FVC: forced expiratory volume in three seconds as a ratio of the forced vital capacity; FEF_25-75_: Mean forced expiratory flow rate between 25% and 75% of the forced vital capacity. Cardiovascular disease: self-reported history of heart disease or stroke. Covariates in the adjusted model: sex, education level, body mass index, smoking status, smoking pack-years and spirometric restriction. Dyspnoea measured according to mMRC Dyspnoea scale: 0-1= minimal/no breathlessness, ≥2= significant breathlessness. Chronic cough: cough on most days for 3 months each year. Chronic Phlegm: Phlegm on most days three months each year. Wheeze: Wheezing or whistling in the chest at any time in the last 12 months. Covariates in the adjusted model: sex, education level, body mass index, smoking status, smoking pack-years, passive smoking, occupational exposure to dust, use of solid fuels for cooking/heating for >6 months in a lifetime, reported doctor-diagnosed or history of tuberculosis, spirometric restriction family history of COPD, and for Dyspnoea addition of CVD. I2 values of 0%, 25%, 50%, and 75% considered no, low, moderate, and high heterogeneity. P-value represents significance for Chi-squared test for heterogeneity of pooled estimates, p<0.05= significant.*
